# Association between patient-reported outcomes and accelerometer-based activity in elderly patients 12 months after proximal humerus fracture: Results from two randomized controlled trials

**DOI:** 10.1371/journal.pone.0352276

**Published:** 2026-08-21

**Authors:** Aleksi Reito, Rasmus Liukkonen, Antti P. Launonen, Helle K. Østergaard, Inger Mechlenburg, Tore Fjalestad, Marianne Toft, Ville Mattila

**Affiliations:** 1 Center for Musculoskeletal diseases, Tampere University Hospital, Faculty of Medicine and Health Technology, Tampere University, Tampere, Finland; 2 Department of Orthopaedic Surgery, Viborg Regional Hospital, Denmark; 3 Department of Orthopaedics, Aarhus University Hospital, Aarhus, Denmark; 4 Department of Clinical Medicine, Aarhus University, Aarhus, Denmark; 5 Research Center for Prevention and Health Promotion, VIA University College, Aarhus, Denmark; 6 Department of Orthopaedic Surgery, Division of Surgery, Oslo University Hospital, Oslo, Norway; University Hospital Zurich, SWITZERLAND

## Abstract

**Aims:**

Patient-reported outcome measures (PROMs) represent outcomes important to patients and are subjective rather than objective measures like mortality or reoperations. Their use has, however, limitations. We aimed to investigate the association between PROMS and accelerometer-based movement in older adults with proximal humerus fractures.

**Methods:**

This is a secondary analysis of two previously conducted randomized controlled trials. The first trial included patients aged 60 years or more with any proximal humerus fracture allocated to nonoperative treatment or operative treatment. The second trial included patients aged 60 years or more with a 2-part fracture allocated to different physiotherapy regimes. A subgroup of patients in these trials wore tri-axial accelerometers in both their upper extremities for 4 days at the 1-year follow-up. Functional outcome was measured with Disability of Arm, Shoulder and Hand (DASH), Constant score, and 15D quality of life questionnaire. Ordinary least squares regression was used to estimate the association between PROMs and accelerometer-based activity.

**Results:**

The final study cohort included 100 patients with a mean age of 73 years. Patients were predominantly females (86%) with a 2-part fracture (65%). All activity level categories (inactivity, light, moderate) had a weak association with DASH when adjusted for baseline variables. Based on the confidence intervals, none of the regression coefficients have statistical significance for the association. Similar findings were seen for 15D quality-of-life measurements.

**Conclusions:**

Our results show that, in older adults, commonly used PROMs have a negligible and uncertain association with objectively measured upper extremity activity 1 year after a proximal humerus fracture.

**Level of Evidence:** III

## Introduction

Proximal humeral fractures (PHF) are common musculoskeletal injuries, especially in the elderly [1,2]. Treatment options include various surgical techniques and nonoperative methods with rehabilitation. Recent studies suggest non-surgical treatment is not inferior to surgical management [3–7]. These conclusions are often based on patient-reported outcome measures (PROMs).

PROMs are tools used to report outcomes important to patients and are subjective rather than objective measures like mortality or adverse events. Concerns have been raised regarding the possibility of misleading conclusions based solely on PROMs [1]. Designed to measure specific factors with standardized questions, PROMs yield a numerical score representing the patient’s condition. However, these standardized questions limit the scope of conditions that can be measured.

PROMs may not correlate with functional recovery [8], so objective data on function is also warranted. For hip and knee conditions, physical activity has been measured with accelerometers in several studies, and reference material exists [2–5,9]. In recent years, accelerometer-based activity measurement has gained interest. They measure the rate of change in velocity, or acceleration, caused by movement or gravitational forces. Most wearable accelerometers measure along three axes—X, Y, and Z—corresponding to movement in three-dimensional space. This allows them to capture complex, multi-directional motion. Accelerometers detect changes in body movement to count steps and measure walking speed, distance traveled, and overall activity level, which are essential metrics for monitoring recovery progress, particularly in patients with fractures.

However, the literature on physical activity or movement measured with accelerometers in shoulder conditions is limited. Accelerometer-based data would provide a more objective evaluation of functional recovery than PROMs alone. A recent study was unable to show associations between accelerometer-based movement and common PROMs in patients having undergone a reverse total shoulder arthroplasty [8]. No study has investigated patients with acute musculoskeletal trauma.

Accelerometer-based data have not been used to evaluate the functional outcomes of PHF treatment. We aimed to investigate the association between accelerometer-based movement and functional outcomes measured by PROMs in older adults with PHFs.

## Methods

### Study cohorts

This study combines data from two multicentre randomized controlled trials (RCTs) conducted by the NITEP group. Tampere University Hospital ethics committee has approved the study (R10127) [10].

The first trial compared nonoperative and operative treatments in older adults with proximal humerus fractures (NCT01246167). Patient recruitment for this trial began in 2011, and the study protocol and primary results have been published previously.

Patients aged 60 or older with a displaced 2-, 3-, or 4-part proximal humerus fracture were randomly assigned to either nonoperative or operative treatment. The study had two strata:

- Stratum 1: Patients with a 2-part fracture, who were randomly allocated in a 1:1 ratio to either nonoperative treatment or operative treatment with a locking-compression plate (LCP).
- Stratum 2: Patients with 3- or 4-part fractures, who were randomly allocated in a 1:1:1 ratio to one of three treatments: nonoperative treatment, operative treatment with an LCP, or operative treatment with hemiarthroplasty (HA).

The second trial compared physiotherapist-supervised and unsupervised home-based exercises for older adults aged 60 or older with a 2-part proximal humerus fracture (NCT03498859). Patient recruitment for this trial began in 2018 [11].

### Outcome measurements

**Fig 1:**
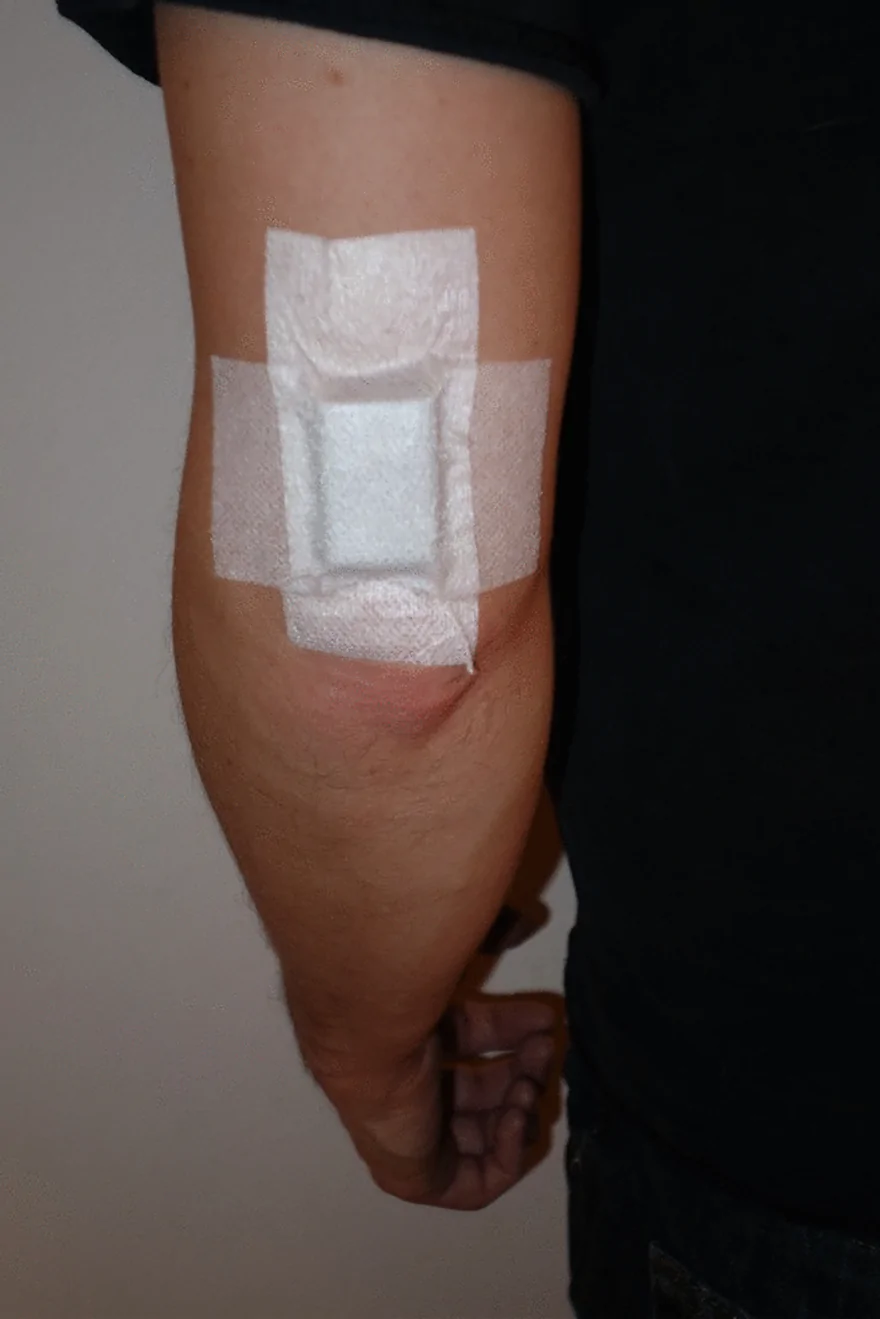
Placement of the accelerometer.

The primary outcome in both trials was the DASH, with the Constant score, 15D quality of life questionnaire as secondary outcomes. In addition, information on the participants’ upper arm activity was obtained with tri-axial accelerometry. Patients wore triaxial accelerometers on both upper arms for four consecutive days at 1-year follow-up. The accelerometers used were the AX3 model from Axivity Ltd. (Newcastle, UK), which measured accelerations in three dimensions at a frequency of 50 Hz and a range of 16 g. A physiotherapist or a nurse attached an accelerometer to the Heuter triangle of the elbow on each arm (Fig 1). When the accelerometers were returned, data were downloaded using OMGUI Configuration and Analysis Tool (Version 1.0.0.43, Newcastle, UK).

In the first trial, accelerometer data were collected starting in 2015, while accelerometer measurements were part of the initial protocol in the second trial, beginning in 2018.

For the present study, accelerometer data were retrieved from patients recruited at Tampere University Hospital in Finland and Viborg Regional Hospital in Denmark, where accelerometer data were explicitly collected. Data were not collected at the other participating sites in the initial trials. Comparison between patients included in this secondary analysis and those who were not included, is shown in S1 File.

At baseline, the common variables recorded included age, gender, fracture morphology, treatment method, body mass index (BMI), and hand dominance. These variables were included in the study as covariates. The associations between baseline variables and inclusion in the study analyses were assessed using logistic regression and are presented in S1 File (SFile1).

### Participants

**Fig 2:**
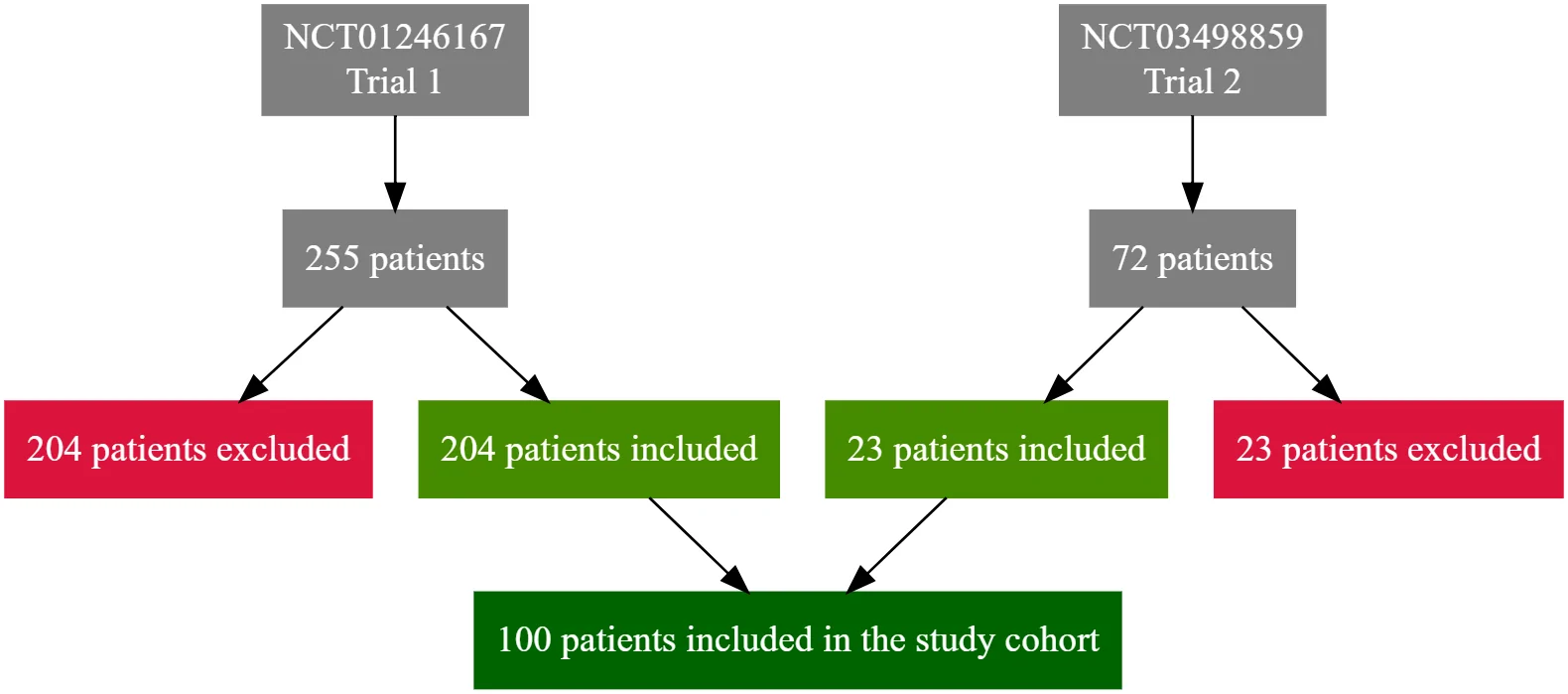
Flow chart of the selection of the study population.

In trial 1, 51 patients participated in the accelerometer measurements. In trial 2, accelerometer measurements were part of the protocol, although 23 patients did not contribute with data due to technical issues or loss to follow-up. As a result, 49 patients from trial 2 participated in the accelerometer measurements, bringing the total number of patients eligible for this secondary analysis to 100 (Fig 2).

### Statistical analysis

Raw data from accelerometers was extracted and analyzed with an open-source R algorithm (GGIR) to estimate time spent in minutes per day in inactivity, light activity and moderate-to-vigorous activity [12]. To date, no externally validated thresholds to categorize raw data to these domains have been published. We used two different threshold value combinations based on wrist measurements. The first extraction was done using the threshold proposed by Dibben et al. [13] Their study included 22 heart failure adults with a mean age of 71 years. Second extraction was done using threshold values proposed by Sanders et al. [14] Their study included 34 healthy older adults with a mean age of 69 years. These studies reported clearly different cut-off values (light: 18, moderate, 45 and light 57: moderate 104, respectively).

Age, gender, fracture morphology, body mass index (BMI) and hand dominance, were collected at baseline in both trials and included in the analyses. The association between accelerometer-based activity and PROMs were assessed with linear regression. QQ plots were evaluated to see that the linear model was suitable (Supplementary figures 1–12 in S1 File). We performed univariable and multivariable analyses, including all aforementioned baseline variables as covariates. For each activity category (e.g., inactivity, light, moderate-to-vigorous), separate linear regression models were fitted with the PROM as the dependent variable and time spent in that activity category (minutes/day) as the independent variable. Adjusted models additionally included predefined covariates (age, sex, treatment allocation, BMI, dominance, and fracture characteristics), while retaining the activity variable as the main exposure [15]. To aid interpretation, activity time was scaled per 100 minutes. Predicted mean PROM values were estimated for each activity category from multivariable models. Analyses were in done with RStudio and rms package.

## Results

**Fig 3:**
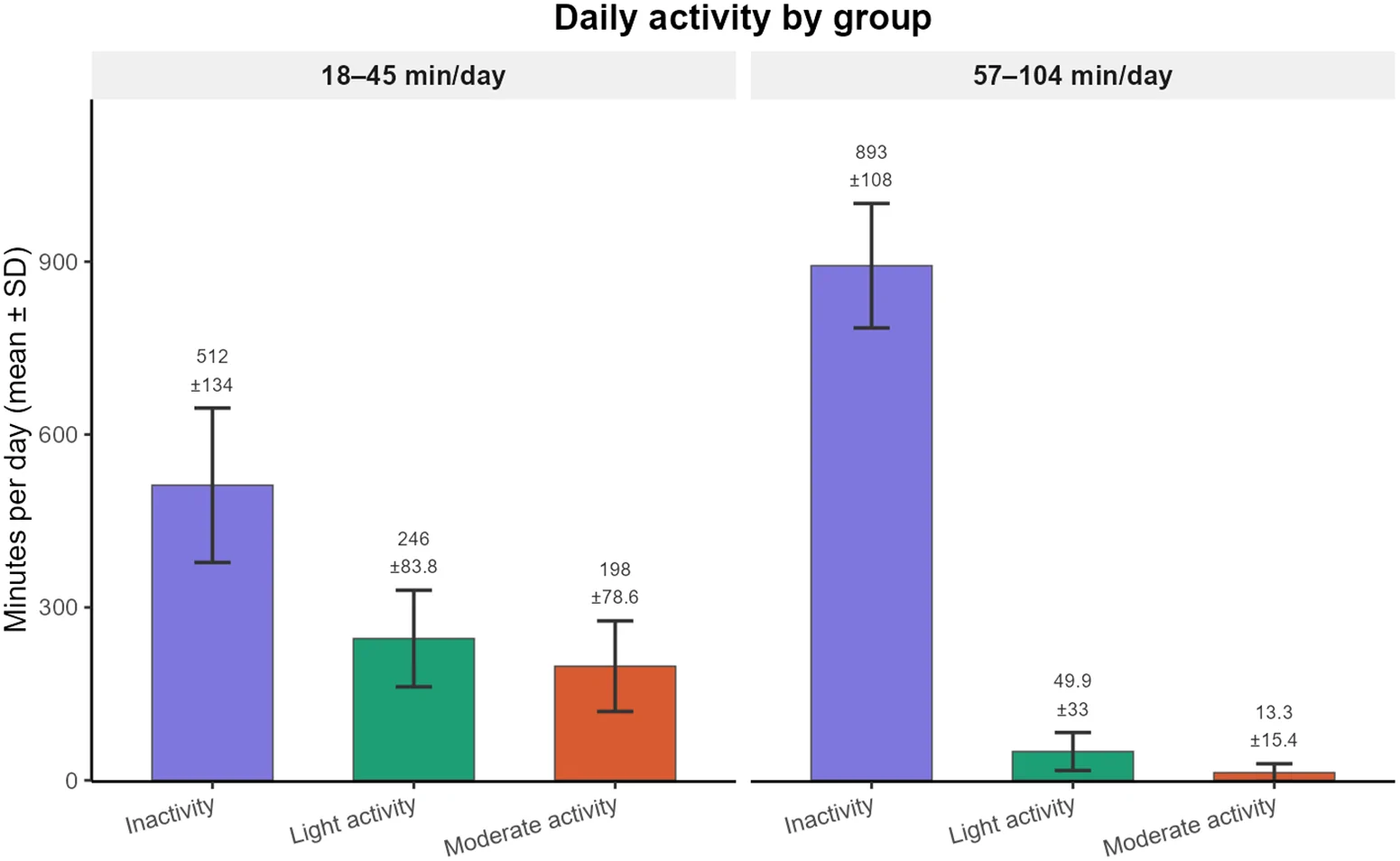
Distribution of the time (minutes) spent in each activity level in the injured arm using two different thresholds. a) Results based on threshold values proposed by Dibben et al. b) Results based on threshold values proposed by Sanders et al.

The final study cohort included 100 patients with a mean age of 73 (SD 7.3) (Table 1). Patients were predominantly females (86%) with a 2-part fracture (65%). Of those, 67 were treated nonoperatively, 27 with a plate fixation, and 6 with a hemiprosthesis. At 12 months, the injured arm, compared to the uninjured arm, spent more time in inactivity and less time in light or moderate physical activity, based on lower cut-off values. For higher cut-offs, the difference did not reach statistical significance (see Supplementary File 1). Fig 3 shows the time spent in each activity level using two different cut-offs for the injured arm.

**Table 1:** Patient characteristics.

Characteristic	Trial 1N = 51^*1*^	Trial 2N = 49^*1*^
**Mean Age (SD)**	72.8 (7.5)	73.3 (7.3)
**Mean BMI (SD)**	27.2 (4.7)	26.7 (5.1)
**Treatment**		
Nonop	18 (35%)	49 (100%)
Plate	27 (53%)	0 (0%)
Prosthesis	6 (12%)	0 (0%)
**Fracture morphology**		
2-part	16 (31%)	49 (100%)
3- or 4-part	35 (69%)	0 (0%)
**Gender**		
Male	5 (10%)	8 (16%)
Female	46 (90%)	41 (84%)
**Dominant hand injured**	25 (49%)	25 (51%)

**Fig 4:**
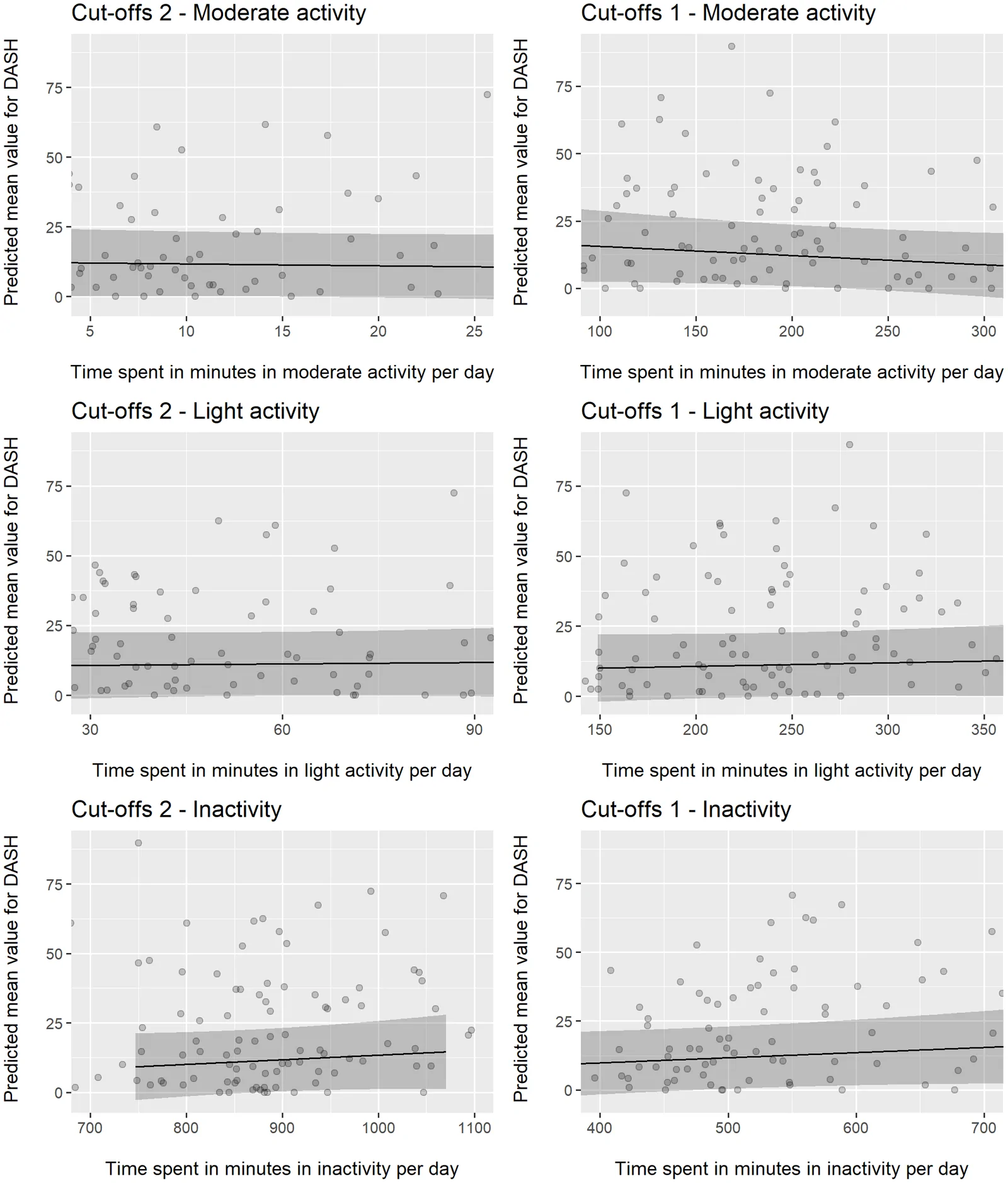
Predicted median values for DASH against varying levels of activity time.

**Fig 5:**
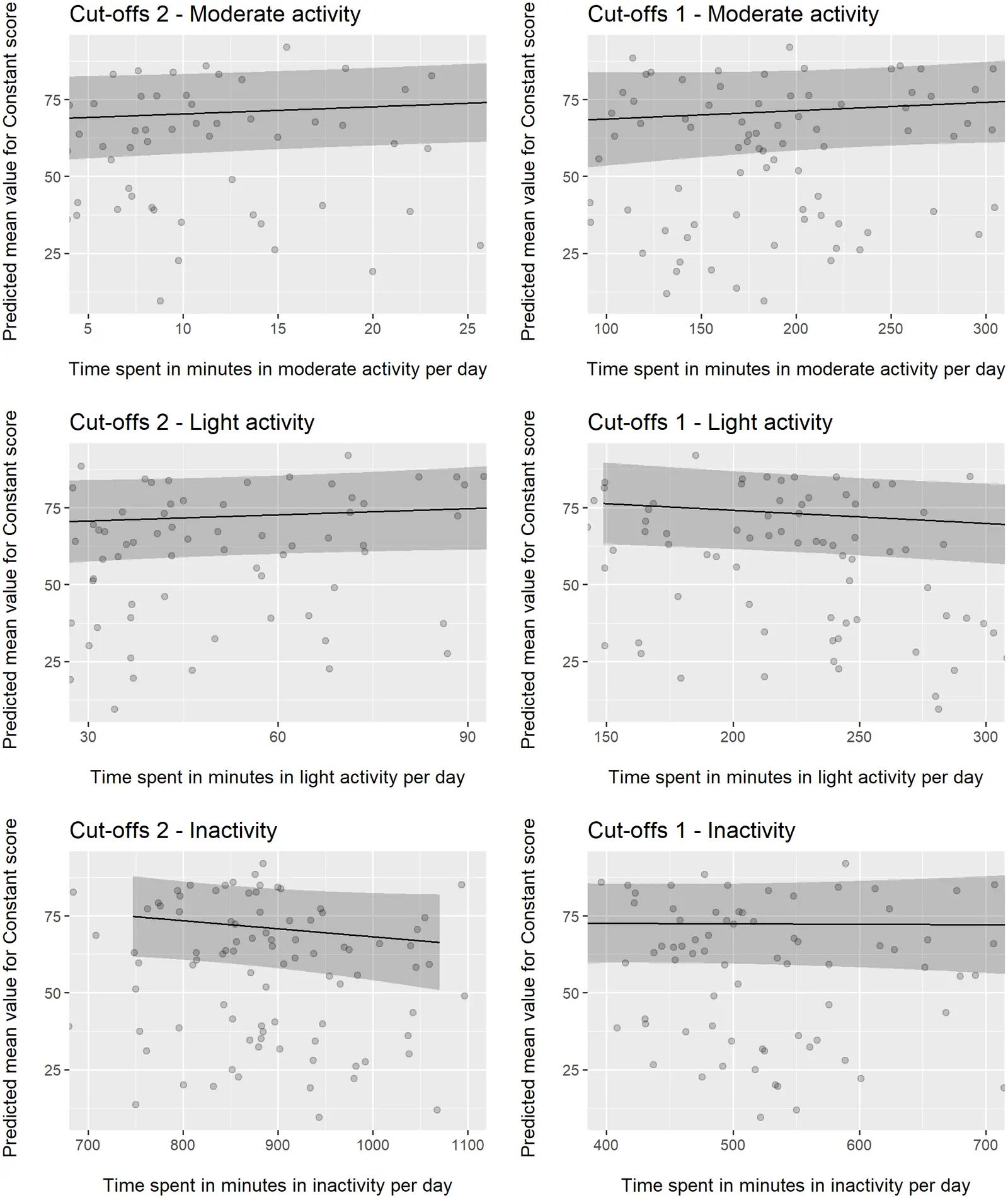
Predicted median values for Constant score against varying levels of activity time.

In the univariable analysis, time spent in inactivity for the injured arm had a weak association with the DASH scores at 12 months for both cut-off values (Table 2). When other baseline variables were included, the effects remained imprecise and non-significant, i.e., had wide confidence intervals. A β of −3.4 for moderate-to-vigorous activity indicates that an increase of 100 minutes per day in this activity category is associated with a 3.4-point lower DASH score (i.e., better function). Results were similar for light activity. For moderate-to-vigorous activity, the univariable analysis showed an association with DASH scores, but these effects were imprecise after adjustment. Predicted adjusted mean values are shown in Fig 4. Wide prediction intervals depict the uncertainty related to the association. Results for Constant score similar as for DASH (Fig 5, Table 3).

**Table 2:** Association between time spent (per 100-minute increase) in each activity category and DASH at 12 months. β represents the change in DASH associated with a 100-minute increase in time spent in the given activity category, based on linear regression models fitted separately for each activity category. Adjusted models include age, sex, treatment allocation, BMI, dominance, and fracture characteristics. As total daily time is constrained, increased time in one activity category may reflect reduced time in another. 95% confidence intervals are shown in brackets.

	Unadjusted β for activity	p-value	Adjusted β for activity	p-value
Inactivity 1	2.27 (−0.89 - 5.4)	0.16	1.87 (−1.1 - 4.8)	0.21
Inactivity 2	2.56 (−1.3 - 6.4)	0.19	1.66 (−1.8 - 5.1)	0.33
Light 1	2.66 (−2.3 - 7.7)	0.29	1.25 (−3.3 - 5.8)	0.59
Light 2	0.37 (−12 - 13)	0.95	1.69 (−10.0–14.0)	0.78
Moderate-to-vigorous 1	−5.7 (−11 - −0.51)	0.03	−3.42 (−8.6 - 1.8)	0.19
Moderate-to-vigorous 2	−33.3 (−60 - −6.7)	0.01	−6.79 (−34 − 20)	0.62

**Table 3:** Association between time spent (per 100-minute increase) in each activity category and Constant score at 12 months. β represents the change in Constant score associated with a 100-minute increase in time spent in the given activity category, based on linear regression models fitted separately for each activity category. Adjusted models include age, sex, treatment allocation, BMI, dominance, and fracture characteristics. As total daily time is constrained, increased time in one activity category may reflect reduced time in another. 95% confidence intervals are shown in brackets.

	Unadjusted β for activity	p-value	Adjusted β for activity	p-value
Inactivity 1	−1.57 (−4.9 - 1.7)	0.34	−1.8 (−5.2 - 1.6)	0.30
Inactivity 2	−3.77 (−7.8 - 0.23)	0.06	−2.32 (−6.2 - 1.6)	0.34
Light 1	−1.8 (−6.9 - 3.2)	0.48	0.74 (−4.2 - 5.7)	0.76
Light 2	11.2 (−1.5 - 24)	0.08	1.06 (−12 - 14)	0.87
Moderate-to-vigorous 1	2.48 (−2.9 - 7.9)	0.36	−0.36 (−6.3 - 5.5)	0.90
Moderate-to-vigorous 2	24.1 (−3.6 - 52)	0.09	10 (−21 - 41)	0.52

**Fig 6:**
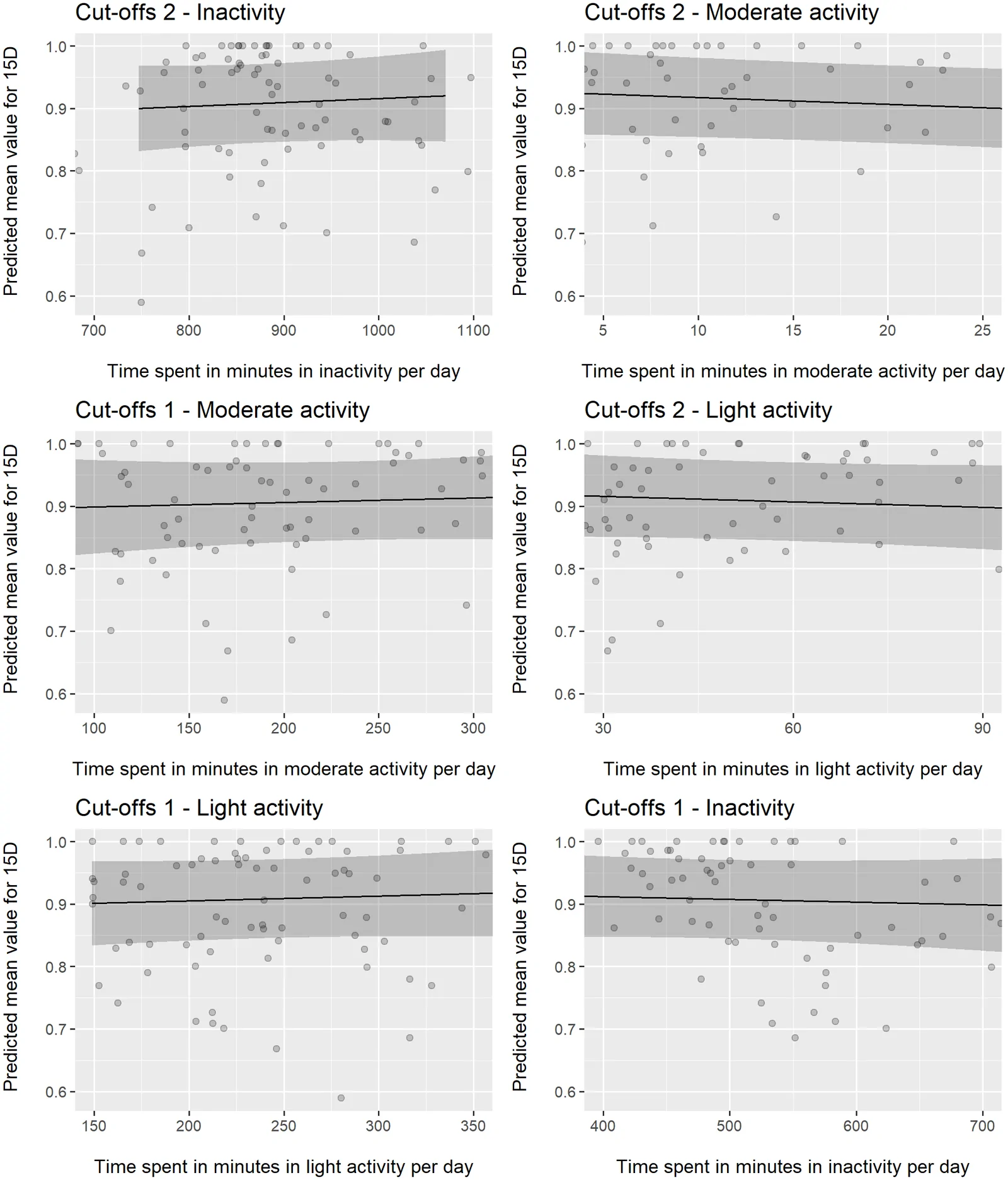
Predicted adjusted median values for 15D against varying levels of activity time.

For the 15D quality of life measurement, time spent in each activity category had a very weak and imprecise association in both univariable and multivariable models (Table 4). The predicted adjusted mean values are shown in Fig 6, with wide prediction intervals highlighting the uncertainty of the association.

**Table 4:** Association between time spent (per 100-minute increase per day) in each activity category and 15D at 12 months. β represents the change in 15D associated with a 100-minute increase in time spent in the given activity category, based on linear regression models fitted separately for each activity category. Adjusted models include age, sex, treatment allocation, BMI, dominance, and fracture characteristics. As total daily time is constrained, increased time in one activity category may reflect reduced time in another. 95% confidence intervals are shown in brackets.

Activity category	β (per 100 min/day increase in activity)	p-value	Adjusted β* (per 100 min/day increase)	p-value
Inactivity 1	−0.013 (−0.028 - 0.0028)	0.11	−0.0044 (−0.022 - 0.013)	0.61
Inactivity 2	−0.0020 (−0.022 - 0.018)	0.84	0.0063 (−0.014 - 0.026)	0.54
Light 1	0.0089 (−0.016 - 0.034)	0.48	0.0077 (−0.018 - 0.033)	0.55
Light 2	−0.0085 (−0.072 - 0.055)	0.79	−0.029 (−0.098 - 0.04)	0.40
Moderate-to-vigorous 1	0.023 (−0.003 - 0.049)	0.08	0.0072 (−0.023 - 0.037)	0.63
Moderate-to-vigorous 2	0.055 (−0.079 - 0.19)	0.42	−0.110 (−0.26 - 0.045)	0.16

*Adjusted for age, sex, treatment group, BMI, dominance, and fracture characteristics.

## Discussion

The present study estimated the association between PROMs and the objectively measured accelerometer-based movement. Based on our analyses, PROMs have, at best, weak and uncertain association with objectively measured upper extremity activity. Given the heterogeneity of recovery patterns and the limited statistical power for detecting modest associations, these analyses should be interpreted as exploratory. A few authors have examined the associations between subjectively reported outcomes and objectively measured activity levels. Luna et al. compared PROMs of the total hip or knee arthroplasty patients to the objectively assessed physical activity [16]. As a result, they reported that the association between these two measurement methods is close to zero, as no significant correlation was present. Edwards et al. examined the association between accelerometer-based activity and PROMs in patients with total shoulder replacement [8]. While accelerometer-based activity increased postoperatively, it did not show an association with PROMs. Birch et al. demonstrated that patient-reported pain catastrophizing in knee osteoarthritis did not affect objectively measured physical activity before total knee arthroplasty, nor at 3 and 12 months post-surgery [17]. Most recently, Yocum et al. evaluated the association between accelerometer-measured physical activity and PROMs after total knee arthroplasty [18]. As a result of their analyses, they also reported that the correlation between objective and subjective measures is weak at best. In conclusion, they stated that it might be possible that the objectively measured outcomes and subjective outcomes might measure two different dimensions in the rehabilitation process. Hence, these should be evaluated separately.

Similar results were seen in our analyses, as the associations were weak at best. These results might be explained by the fact that the nature of the PROMs is restricted to certain dimensions. Hence, they only measure prespecified conditions rather than assessing the global improvement of the patient. Although PROMs are based on specific questions, their weak correlation with objectively measured activity levels suggests that additional instruments may complement PROMs when evaluating different dimensions of recovery. The results from our analyses are consistent with the hypothesis that these methods may capture partly distinct dimensions of recoveryof subjective and objective evaluation, as each warrants their own assessment when assessing the individual patient’s rehabilitation. Similar conclusions have also been reported as early as 2011. Still, to date, objectively measured activity levels are not routinely used to evaluate rehabilitation due to their higher cost and complexity compared to PROMs. Taken together, these studies and our findings consistently suggest that associations between subjective and objective measures are weak. However, these results should be interpreted with caution, as variability in patient populations, measurement methods, and follow-up time points may contribute to heterogeneity across studies.

The accelerometer-based measurement of activity might differ significantly from subjectively reported measures. For example, a patient may not have performed certain activities, even though they can, leading to inaccurate responses on subjective measurements. The questionnaire response would indicate the “best-case scenario” of the patient´s ability. In other words, if the patient does not perform the activities that the subjective outcome measures evaluate, the answers may not be accurate, affecting the interpretation of these instruments. In part, this may be explained by recall bias. Conversely, it is also possible that the patient might overestimate their abilities if certain activities have not been performed. While objective measures are essential for standardised assessment, treatment decisions must ultimately reflect patient-perceived recovery and quality of life, as these determine the lived impact of the outcome.

DASH is a subjective assessment of patient-reported outcomes, as the Constant score also includes objective measurements. Hence, we were surprised that the correlation with accelerometer-based activity measurements was also weak for the Constant score. As a comprehensive assessment of shoulder function, the Constant score evaluates key recovery parameters, including pain, strength, range of motion, and activities of daily living. While it may not directly reflect real-world activity levels, its structured approach ensures a thorough examination of a patient’s functional capacity. One of the Constant score’s main advantages is its ability to capture essential aspects of shoulder performance beyond mere movement. Strength and ROM, integral components of the score, are crucial for long-term recovery and return to full function. However, similar to DASH, these objective measurements reflect the “best-case scenario” and straightforwardly describe what the patient does or performs in everyday life. An important limitation of the Constant score is that strength measurements can only be carried out if the patient can elevate the shoulder to 90 degrees. If not, the strength score is recorded as 0. Consequently, a low total Constant score does not necessarily reflect a low activity level, which may help explain the weak correlation between the Constant score and accelerometer-based activity measurements.

Regardless of the lack of association, accelerometer-based activity may provide additional information on real-world activity patterns, although their role in evaluating recovery remains to be established after injuries or elective surgeries. Still, further research is needed to elaborate the utility of accelerometer-based activity measures. PROMs have their limitations as discussed by numerous authors. Regardless of their shortcomings, they, however, provide a very crucial outcome tool as functional PROMs are known to correlate with quality-of-life measurements and treatment satisfaction. Moreover, based on functional PROMs, different thresholds for treatment failure and patient-accepted symptoms states have been established, which are very valuable to assess treatment effectiveness. Similar analytical approaches are needed for objective activity measurement for give further insights of their role.

The present analyses are exploratory and should be interpreted with caution. As we used PROMs as a part of the analyses, the limitations of these, such as the ceiling effect, might be present. However, as it has been previously reported that the conclusions based solely on PROMs might be inaccurate, it highlights the need for additional evaluations, such as accelerometer-based measurements. Furthermore, as the patients wore the accelerometer for short periods of time rather than continuously through the rehabilitation, it might be possible that the measurements might have been prone to bias, as for example, the patient might have been especially active during the measurement periods. Activity was measured at a single time point (12 months) over a short monitoring period of four days, which limits the ability to capture longitudinal recovery patterns or habitual activity over longer periods. The present findings are restricted to the association between PROMs and objectively measured activity at 12 months and should not be extrapolated to earlier phases of recovery or to long-term activity patterns. On the other hand, the accelerometer-based data is a remarkable advantage of the present study, as no previous trial has examined the associations between the objectively measured outcomes to the subjectively measured outcomes after proximal humeral fractures. One limitation was also that we measured activity only at one time-point. Repeated measures could theoretically give more insight to the healing process. Finally, accelerometer-based activity analyses are sensitive to threshold how raw data is labelled. No externally validated thresholds exist. We mitigated this problem using two different threshold value sets.

## Conclusion

In this study, objectively measured upper extremity activity at a single time point (12 months) after proximal humerus fracture showed a weak and uncertain association with PROMs, including DASH, Constant score, and 15D. These findings suggest that subjective and objective measures may reflect different, only partly overlapping aspects of recovery rather than a single underlying construct. However, given the exploratory nature of the analyses, heterogeneity in recovery patterns, and limitations related to both accelerometry and PROMs, the results should be interpreted with caution and restricted to the studied time point. Accelerometer-based activity measurement does not appear to provide a standalone solution for assessing recovery, but rather offers complementary information that requires careful interpretation alongside established outcome measures. Its role should be clarified in studies incorporating longitudinal measurements, clinically meaningful thresholds, and links to patient-centered outcomes.

## Supporting information

S1 File SFile1. (DOCX)

S1 Data SData1. (XLSX)
