# Supplementary material for: Association between patient-reported outcomes and accelerometer-based activity in elderly patients 12 months after proximal humerus fracture: Results from two randomized controlled trials
Source: PLoS One. 2026 Aug 21;21(8):e0352276. doi: 10.1371/journal.pone.0352276 (PMC13496457; doi:10.1371/journal.pone.0352276)
Supplement: Supplementary file 1 [file pone.0352276.s001.docx]

Supplementary analysis 1:

Supplementary analysis 2:

```
Welch Two Sample t-test

data:  data$axIN1845 and data$axINH1845
t = 3.382, df = 41.522, p-value = 0.001579
alternative hypothesis: true difference in means is not equal to 0
95 percent confidence interval:
  46.62563 184.72158
sample estimates:
mean of x mean of y
 511.7182  396.0446


	Welch Two Sample t-test

data:  data$axLIG1845 and data$axLIGH1845
t = -2.0612, df = 38.007, p-value = 0.04617
alternative hypothesis: true difference in means is not equal to 0
95 percent confidence interval:
 -101.9899441   -0.9196688
sample estimates:
mean of x mean of y
 245.6299  297.0847


	Welch Two Sample t-test

data:  data$axMOD1845 and data$axMODH1845
t = -3.9622, df = 39.258, p-value = 0.0003043
alternative hypothesis: true difference in means is not equal to 0
95 percent confidence interval:
 -131.7023  -42.6929
sample estimates:
mean of x mean of y
 198.2973  285.4949


	Welch Two Sample t-test

data:  data$axIN57104 and data$axINH57104
t = -0.27943, df = 57.442, p-value = 0.7809
alternative hypothesis: true difference in means is not equal to 0
95 percent confidence interval:
 -45.33924  34.23372
sample estimates:
mean of x mean of y
 892.5541  898.1069


	Welch Two Sample t-test

data:  data$axLIG57104 and data$axLIGH57104
t = -1.4439, df = 36.352, p-value = 0.1573
alternative hypothesis: true difference in means is not equal to 0
95 percent confidence interval:
 -37.732797   6.342753
sample estimates:
mean of x mean of y
 49.92346  65.61848


	Welch Two Sample t-test

data:  data$axMOD57104 and data$axMODH57104
t = -0.52656, df = 47.752, p-value = 0.6009
alternative hypothesis: true difference in means is not equal to 0
95 percent confidence interval:
 -8.415477  4.922814
sample estimates:
mean of x mean of y
 13.27011  15.01645
```

Supplementary Table 1:

Supplementary Figure 1: QQ plot of studentized residuals from the multivariable regression model examining DASH and Inactivity 1

Supplementary Figure 2: QQ plot of studentized residuals from the multivariable regression model examining 15D QoL and Inactivity 1

Supplementary Figure 3: QQ plot of studentized residuals from the multivariable regression model examining DASH and Inactivity 2

Supplementary Figure 4: QQ plot of studentized residuals from the multivariable regression model examining 15D QoL and Inactivity 2

Supplementary Figure 5: QQ plot of studentized residuals from the multivariable regression model examining DASH and Light activity 1

Supplementary Figure 6: QQ plot of studentized residuals from the multivariable regression model examining 15D QoL and Light activity 1

Supplementary Figure 7: QQ plot of studentized residuals from the multivariable regression model examining DASH and Light activity 2

Supplementary Figure 8: QQ plot of studentized residuals from the multivariable regression model examining 15D QoL and Light activity 2

Supplementary Figure 9: QQ plot of studentized residuals from the multivariable regression model examining DASH and Moderate activity 1

Supplementary Figure 10: QQ plot of studentized residuals from the multivariable regression model examining 15D QoL and Moderate activity 1

Supplementary Figure 11: QQ plot of studentized residuals from the multivariable regression model examining DASH and Moderate activity 2

Supplementary Figure 12: QQ plot of studentized residuals from the multivariable regression model examining 15D QoL and Moderate activity 2
