# Supplementary material for: Association between patient-reported outcomes and accelerometer-based activity in elderly patients 12 months after proximal humerus fracture: Results from two randomized controlled trials
Source: PLoS One. 2026 Aug 21;21(8):e0352276. doi: 10.1371/journal.pone.0352276 (PMC13496457; doi:10.1371/journal.pone.0352276)
Supplement: Peer review history

# Peer review: Decision Letter 0

**DOI:** 10.1371/journal.pone.0352276.r001

**Authors:** Hans-Peter Simmen

9 Feb 2026

Dear Dr. Reito,

Thank you for submitting your manuscript to PLOS ONE. After careful consideration, we feel that it has merit but does not fully meet PLOS ONE’s publication criteria as it currently stands. Therefore, we invite you to submit a revised version of the manuscript that addresses the points raised during the review process.

Both reviewers are experts in the field and give well-intentioned recommendations to improve your manuscript. Please, follow their ideas.

Please submit your revised manuscript by Mar 26 2026 11:59PM. If you will need more time than this to complete your revisions, please reply to this message or contact the journal office at plosone@plos.org. When you're ready to submit your revision, log on to https://www.editorialmanager.com/pone/ and select the 'Submissions Needing Revision' folder to locate your manuscript file.

- A letter that responds to each point raised by the academic editor and reviewer(s). You should upload this letter as a separate file labeled 'Response to Reviewers'.
- A marked-up copy of your manuscript that highlights changes made to the original version. You should upload this as a separate file labeled 'Revised Manuscript with Track Changes'.
- An unmarked version of your revised paper without tracked changes. You should upload this as a separate file labeled 'Manuscript'.

If you would like to make changes to your financial disclosure, please include your updated statement in your cover letter. Guidelines for resubmitting your figure files are available below the reviewer comments at the end of this letter.

If applicable, we recommend that you deposit your laboratory protocols in protocols.io to enhance the reproducibility of your results. Protocols.io assigns your protocol its own identifier (DOI) so that it can be cited independently in the future. For instructions see: https://journals.plos.org/plosone/s/submission-guidelines#loc-laboratory-protocols. Additionally, PLOS ONE offers an option for publishing peer-reviewed Lab Protocol articles, which describe protocols hosted on protocols.io. Read more information on sharing protocols at https://plos.org/protocols?utm_medium=editorial-email&utm_source=authorletters&utm_campaign=protocols.

We look forward to receiving your revised manuscript.

Kind regards,

Hans-Peter Simmen, M.D., Professor of Surgery

Academic Editor

PLOS One

Journal requirements:

When submitting your revision, we need you to address these additional requirements.

1. Please ensure that your manuscript meets PLOS ONE's style requirements, including those for file naming. The PLOS ONE style templates can be found at

https://journals.plos.org/plosone/s/file?id=wjVg/PLOSOne_formatting_sample_main_body.pdf and

https://journals.plos.org/plosone/s/file?id=ba62/PLOSOne_formatting_sample_title_authors_affiliations.pdf

2. We note that you have indicated that there are restrictions to data sharing for this study. For studies involving human research participant data or other sensitive data, we encourage authors to share de-identified or anonymized data. However, when data cannot be publicly shared for ethical reasons, we allow authors to make their data sets available upon request. For information on unacceptable data access restrictions, please see http://journals.plos.org/plosone/s/data-availability#loc-unacceptable-data-access-restrictions.

Before we proceed with your manuscript, please address the following prompts:

a) If there are ethical or legal restrictions on sharing a de-identified data set, please explain them in detail (e.g., data contain potentially identifying or sensitive patient information, data are owned by a third-party organization, etc.) and who has imposed them (e.g., a Research Ethics Committee or Institutional Review Board, etc.). Please also provide contact information for a data access committee, ethics committee, or other institutional body to which data requests may be sent.

b) If there are no restrictions, please upload the minimal anonymized data set necessary to replicate your study findings to a stable, public repository and provide us with the relevant URLs, DOIs, or accession numbers. Please see http://www.bmj.com/content/340/bmj.c181.long for guidelines on how to de-identify and prepare clinical data for publication. For a list of recommended repositories, please see https://journals.plos.org/plosone/s/recommended-repositories. You also have the option of uploading the data as Supporting Information files, but we would recommend depositing data directly to a data repository if possible.

Please update your Data Availability statement in the submission form accordingly.

If the reviewer comments include a recommendation to cite specific previously published works, please review and evaluate these publications to determine whether they are relevant and should be cited. There is no requirement to cite these works unless the editor has indicated otherwise.

[Note: HTML markup is below. Please do not edit.]

Reviewers' comments:

Reviewer's Responses to Questions

**Comments to the Author**

1. Is the manuscript technically sound, and do the data support the conclusions?

Reviewer #1: Yes

Reviewer #2: Yes

**********

2. Has the statistical analysis been performed appropriately and rigorously?

Reviewer #1: Yes

Reviewer #2: Yes

**********

3. Have the authors made all data underlying the findings in their manuscript fully available?

The PLOS Data policy

Reviewer #1: Yes

Reviewer #2: Yes

**********

4. Is the manuscript presented in an intelligible fashion and written in standard English?

Reviewer #1: Yes

Reviewer #2: Yes

**********

Reviewer #1: Summary

This manuscript investigates the association between patient-reported outcome measures (PROMs) and objectively measured upper-extremity activity using triaxial accelerometry in older adults following proximal humeral fracture. The study is based on a secondary analysis of data from two randomized controlled trials and addresses an important methodological question regarding outcome assessment in musculoskeletal research.

The manuscript is clearly written, the methods are largely transparent, and the analyses are appropriately cautious in reporting uncertainty. The finding of weak and imprecise associations between PROMs and accelerometer-based activity is consistent with prior literature in other orthopedic populations and is of interest from a measurement and outcomes-research perspective.

However, several methodological issues—particularly regarding selection bias, construct validity of accelerometer thresholds, heterogeneity of the study population, and statistical power—need to be more explicitly addressed. Given PLOS One’s emphasis on scientific validity rather than perceived impact, the manuscript would benefit from improved transparency and more conservative interpretation of results.

________________________________________

Major Comments

1. Selection Bias and Data Availability

o Accelerometer data were collected at only two participating centers, although the parent trials were multicenter.

o The manuscript does not provide a comparison between patients included in this secondary analysis and those without accelerometer data.

Recommendation:

Provide a comparison of baseline characteristics between included and non-included patients or clearly discuss the potential for selection bias and its implications for generalizability.

2. Clinical and Methodological Heterogeneity

o The cohort includes multiple fracture types (2-, 3-, and 4-part), different treatment strategies (nonoperative, plate fixation, hemiarthroplasty), and participants from two trials with distinct interventions.

o While multivariable adjustment was performed, the modest sample size (n = 96) limits the robustness of these models.

Recommendation:

Explicitly discuss the implications of heterogeneity and limited statistical power. Consider clarifying that the analyses are exploratory in nature.

3. Validity of Accelerometer Thresholds

o No validated cut-offs exist for categorizing upper-arm accelerometer data in patients with shoulder pathology.

o The applied thresholds were derived from wrist-worn accelerometers in populations that differ from the present cohort.

Recommendation:

Emphasize this limitation more clearly and avoid framing accelerometer-based activity as a definitive measure of functional recovery.

4. Interpretation of Accelerometer-Based Activity

o Accelerometers measure movement quantity rather than task-specific function, movement quality, or shoulder-specific performance.

o Compensatory movements and passive arm swing cannot be distinguished from purposeful shoulder use.

Recommendation:

Clarify that accelerometer-based measurements represent one dimension of recovery and should be interpreted as complementary to PROMs rather than as a replacement.

5. Timing and Duration of Measurements

o Activity was measured only at a single time point (12 months) and over a short period (four days).

o This limits insight into longitudinal recovery patterns and may be influenced by behavioral modification during monitoring.

Recommendation:

Explicitly acknowledge these limitations and restrict conclusions to the measured time frame.

6. Framing of Conclusions

o The conclusion suggests that PROMs may not reflect “actual functional activity.”

o Given the limitations of both PROMs and accelerometry, this statement may be too strong.

Recommendation:

Reframe conclusions to emphasize weak association and conceptual differences between measurement modalities, consistent with PLOS One’s emphasis on methodological validity rather than clinical inference.

Minor Comments

1. Statistical Reporting

o Clarify whether regression assumptions were formally assessed.

o Consider reporting standardized regression coefficients to facilitate interpretation.

2. Terminology

o Use consistent terminology to distinguish between physical activity, functional capacity, and functional performance.

3. Figures and Tables

o Scatterplots or correlation plots could improve transparency by visualizing the data underlying regression analyses.

o Table captions could more clearly explain the clinical meaning of reported β values.

4. Language and Style

o Minor editing would improve readability by reducing repetition and sentence length in the Discussion.

Reviewer #2: Thank you for letting me review this article that explores the association between direct functional activity measured with an accelerometer and PROMs.

Below are my remarks on the manuscript:

- Table 1 missing. I would appreciate a casual table that displays the patient characteristics. I know that you wrote some demographics in the text but it is important to really see the demographics of the investigated patient cohort.

- A Flowchart displaying the patient inclusion would be helpful. You explain it in the text but it is more helpful to see it displayed in a figure, how many patients were included from both original studies and how many are i.e. lost for follow up.

- Please standardize the numbers in the tables to a defined amount of decimal numbers (i.e. 2-3.). Especially in Table 3 this would be very helpful.

- You mention Figure 2 but further explanation, what 2A and 2B represents, is missing. Overall, I prefer bar-charts over pie-charts... this could also display the standard deviation. I leave it up to you, if you would like to change the style of presentation, but I would suggest it...

-Why was smoking status included in the univariate analysis? Does it affect the activity of the arm in any way? I mean, we are not talking about wound healing problems but functionality, therefore i dont really understand the reason.

- I do not really understand the Tables well enough. I understand that you did a univariate analysis on the association of the activity of the injured arm with different PROMs and two different cutoff definitions. But I cant really wrap my head around the (un)adjusted beta which that displays the change in DASH when the activity level is increased by 100 minutes. So each patient has a certain amount of time in different activity levels and then check if the DASH is better if more time is spent in higher activity? And what specifically is the adjusted beta for activity? I think this requires a little more explanation for the reader to understand properly.

- I think, another limitation might be that the patients might be especially careful with the injured arm if a sensor is attached to it which will later be analyzed by a doctor. I can imagine that they are especially careful, so the doctor does not think, they are doing "too much"...

- Generally, one of the main problems is that thresholds are not validated for the upper extremity. But I like your approach with taking both/all of the known one and comparing them.

- I would appreciate 1-2 sentences discussing the objective vs. subjective outcomes/outcome measures for the patient. I personally think that it is important for the patient to be happy and have a good feeling about the overall outcome because he/she has to live with it. Objective outcome parameter are also necessary but should they overrule the subjective feeling of the patient?

Thank you very much for your work.

**********

what does this mean?). If published, this will include your full peer review and any attached files.

If you choose “no”, your identity will remain anonymous but your review may still be made public.

**Do you want your identity to be public for this peer review?**  For information about this choice, including consent withdrawal, please see our Privacy Policy

Reviewer #1: No

Reviewer #2: No

**********

[NOTE: If reviewer comments were submitted as an attachment file, they will be attached to this email and accessible via the submission site. Please log into your account, locate the manuscript record, and check for the action link "View Attachments". If this link does not appear, there are no attachment files.]

To ensure your figures meet our technical requirements, please review our figure guidelines: https://journals.plos.org/plosone/s/figures

You may also use PLOS’s free figure tool, NAAS, to help you prepare publication quality figures: https://journals.plos.org/plosone/s/figures#loc-tools-for-figure-preparation.

NAAS will assess whether your figures meet our technical requirements by comparing each figure against our figure specifications.

# Author comment: Author response to Decision Letter 1

**DOI:** 10.1371/journal.pone.0352276.r002

3 Jun 2026

Authors’ responses (AR)

Journal requirements:

When submitting your revision, we need you to address these additional requirements.

1. Please ensure that your manuscript meets PLOS ONE's style requirements, including those for file naming. The PLOS ONE style templates can be found at

https://journals.plos.org/plosone/s/file?id=wjVg/PLOSOne_formatting_sample_main_body.pdf and

https://journals.plos.org/plosone/s/file?id=ba62/PLOSOne_formatting_sample_title_authors_affiliations.pdf

2. We note that you have indicated that there are restrictions to data sharing for this study. For studies involving human research participant data or other sensitive data, we encourage authors to share de-identified or anonymized data. However, when data cannot be publicly shared for ethical reasons, we allow authors to make their data sets available upon request. For information on unacceptable data access restrictions, please see http://journals.plos.org/plosone/s/data-availability#loc-unacceptable-data-access-restrictions.

Before we proceed with your manuscript, please address the following prompts:

a) If there are ethical or legal restrictions on sharing a de-identified data set, please explain them in detail (e.g., data contain potentially identifying or sensitive patient information, data are owned by a third-party organization, etc.) and who has imposed them (e.g., a Research Ethics Committee or Institutional Review Board, etc.). Please also provide contact information for a data access committee, ethics committee, or other institutional body to which data requests may be sent.

AR: A de-identified data set can be shared against a reasonable request and research plan. Correspondence can be addressed to the first author.

b) If there are no restrictions, please upload the minimal anonymized data set necessary to replicate your study findings to a stable, public repository and provide us with the relevant URLs, DOIs, or accession numbers. Please see http://www.bmj.com/content/340/bmj.c181.long for guidelines on how to de-identify and prepare clinical data for publication. For a list of recommended repositories, please see https://journals.plos.org/plosone/s/recommended-repositories. You also have the option of uploading the data as Supporting Information files, but we would recommend depositing data directly to a data repository if possible.

AR: Please see the above response.

Please update your Data Availability statement in the submission form accordingly.

If the reviewer comments include a recommendation to cite specific previously published works, please review and evaluate these publications to determine whether they are relevant and should be cited. There is no requirement to cite these works unless the editor has indicated otherwise.

AR: Changed as requested.

Reviewers' comments:

Reviewer's Responses to Questions

Comments to the Author

1. Is the manuscript technically sound, and do the data support the conclusions?

The manuscript must describe a technically sound piece of scientific research with data that supports the conclusions. Experiments must have been conducted rigorously, with appropriate controls, replication, and sample sizes. The conclusions must be drawn appropriately based on the data presented.

Reviewer #1: Yes

Reviewer #2: Yes

________________________________________

2. Has the statistical analysis been performed appropriately and rigorously?

Reviewer #1: Yes

Reviewer #2: Yes

________________________________________

3. Have the authors made all data underlying the findings in their manuscript fully available?

The PLOS Data policy requires authors to make all data underlying the findings described in their manuscript fully available without restriction, with rare exception (please refer to the Data Availability Statement in the manuscript PDF file). The data should be provided as part of the manuscript or its supporting information, or deposited to a public repository. For example, in addition to summary statistics, the data points behind means, medians and variance measures should be available. If there are restrictions on publicly sharing data—e.g. participant privacy or use of data from a third party—those must be specified.

Reviewer #1: Yes

Reviewer #2: Yes

________________________________________

4. Is the manuscript presented in an intelligible fashion and written in standard English?

PLOS ONE does not copyedit accepted manuscripts, so the language in submitted articles must be clear, correct, and unambiguous. Any typographical or grammatical errors should be corrected at revision, so please note any specific errors here.

Reviewer #1: Yes

Reviewer #2: Yes

________________________________________

5. Review Comments to the Author

Please use the space provided to explain your answers to the questions above. You may also include additional comments for the author, including concerns about dual publication, research ethics, or publication ethics. (Please upload your review as an attachment if it exceeds 20,000 characters)

Reviewer #1: Summary

This manuscript investigates the association between patient-reported outcome measures (PROMs) and objectively measured upper-extremity activity using triaxial accelerometry in older adults following proximal humeral fracture. The study is based on a secondary analysis of data from two randomized controlled trials and addresses an important methodological question regarding outcome assessment in musculoskeletal research.

The manuscript is clearly written, the methods are largely transparent, and the analyses are appropriately cautious in reporting uncertainty. The finding of weak and imprecise associations between PROMs and accelerometer-based activity is consistent with prior literature in other orthopedic populations and is of interest from a measurement and outcomes-research perspective.

AR: We thank you for the positive comments.

However, several methodological issues—particularly regarding selection bias, construct validity of accelerometer thresholds, heterogeneity of the study population, and statistical power—need to be more explicitly addressed. Given PLOS One’s emphasis on scientific validity rather than perceived impact, the manuscript would benefit from improved transparency and more conservative interpretation of results.

________________________________________

Major Comments

1. Selection Bias and Data Availability

o Accelerometer data were collected at only two participating centers, although the parent trials were multicenter.

o The manuscript does not provide a comparison between patients included in this secondary analysis and those without accelerometer data.

Recommendation:

Provide a comparison of baseline characteristics between included and non-included patients or clearly discuss the potential for selection bias and its implications for generalizability.

AR: We thank you for your very insightful comment. In the supplementary data, we now assess which variables are associated with a study's inclusion using logistic regression. We also compared baseline variables between included and non-included patients.

2. Clinical and Methodological Heterogeneity

o The cohort includes multiple fracture types (2-, 3-, and 4-part), different treatment strategies (nonoperative, plate fixation, hemiarthroplasty), and participants from two trials with distinct interventions.

o While multivariable adjustment was performed, the modest sample size (n = 96) limits the robustness of these models.

Recommendation:

Explicitly discuss the implications of heterogeneity and limited statistical power. Consider clarifying that the analyses are exploratory in nature.

AR: First paragraph in the discussion is edited as follows: “Based on our analyses, PROMs have, at best, a weak and uncertain association with objectively measured upper extremity activity. Given the heterogeneity of recovery patterns and the limited statistical power for detecting modest associations, these analyses should be interpreted as exploratory.”

3. Validity of Accelerometer Thresholds

o No validated cut-offs exist for categorizing upper-arm accelerometer data in patients with shoulder pathology.

o The applied thresholds were derived from wrist-worn accelerometers in populations that differ from the present cohort.

Recommendation:

Emphasize this limitation more clearly and avoid framing accelerometer-based activity as a definitive measure of functional recovery.

AR: We have rewritten the conclusion and it includes a statement as follows: “Accelerometer-based activity measurement does not appear to provide a turnkey solution for assessing recovery, but rather offers one additional dimension that requires careful interpretation. Its role should be clarified through studies that incorporate longitudinal measurements, define clinically meaningful thresholds, and link objective activity to patient-centered outcomes.”

4. Interpretation of Accelerometer-Based Activity

o Accelerometers measure movement quantity rather than task-specific function, movement quality, or shoulder-specific performance.

o Compensatory movements and passive arm swing cannot be distinguished from purposeful shoulder use.

Recommendation:

Clarify that accelerometer-based measurements represent one dimension of recovery and should be interpreted as complementary to PROMs rather than as a replacement.

AR: We have combined this with the above-mentioned aspects and also added: “Although PROMs are based on specific questions, their weak correlation with objectively measured activity levels suggests that additional instruments may complement PROMs when evaluating different dimensions of recovery”.

5. Timing and Duration of Measurements

o Activity was measured only at a single time point (12 months) and over a short period (four days).

o This limits insight into longitudinal recovery patterns and may be influenced by behavioral modification during monitoring.

Recommendation:

Explicitly acknowledge these limitations and restrict conclusions to the measured time frame.

AR: In the limitations we added: “Activity was measured at a single time point (12 months) over a short monitoring period of four days, which limits the ability to capture longitudinal recovery patterns or habitual activity over longer periods. The present findings are restricted to the association between PROMs and objectively measured activity at 12 months and should not be extrapolated to earlier phases of recovery or to long-term activity patterns”

6. Framing of Conclusions

o The conclusion suggests that PROMs may not reflect “actual functional activity.”

o Given the limitations of both PROMs and accelerometry, this statement may be too strong.

Recommendation:

Reframe conclusions to emphasize weak association and conceptual differences between measurement modalities, consistent with PLOS One’s emphasis on methodological validity rather than clinical inference.

AR: We have now rewritten the conclusion paragraph avoiding too strong statements.

Minor Comments

1. Statistical Reporting

o Clarify whether regression assumptions were formally assessed.

AR: We investigated QQ-plots for our analyses and these are now provided in the Supplement.

o Consider reporting standardized regression coefficients to facilitate interpretation.

AR: We initially considered ordinal and linear regression for the main analyses and preferred the latter due to the interpretability of the regression coefficients. Hence, we prefer the current reporting as they are coefficients with units.

2. Terminology

o Use consistent terminology to distinguish between physical activity, functional capacity, and functional performance.

AR: We thank you for this comment. We have now, e.g., omitted the term “functional activity” and used “physical activity” instead. Function is now related to PROMs.

3. Figures and Tables

o Scatterplots or correlation plots could improve transparency by visualizing the data underlying regression analyses.

AR: Crude values for the activity and outcome variable are now shown in the plots.

o Table captions could more clearly explain the clinical meaning of reported β values.

AR: Other reviewer also commented on this, and the table legends have been edited.

4. Language and Style

o Minor editing would improve readability by reducing repetition and sentence length in the Discussion.

AR: We have made several changes to the discussion, and hopefully, it has improved readability.

Reviewer #2: Thank you for letting me review this article that explores the association between direct functional activity measured with an accelerometer and PROMs.

Below are my remarks on the manuscript:

- Table 1 missing. I would appreciate a casual table that displays the patient characteristics. I know that you wrote some demographics in the text but it is important to really see the demographics of the investigated patient cohort.

AR: Yes of course, to enhance transparency. Table 1 is now provided

- A Flowchart displaying the patient inclusion would be helpful. You explain it in the text but it is more helpful to see it displayed in a figure, how many patients were included from both original studies and how many are i.e. lost for follow up.

AR: Flowchart has been added

- Please standardize the numbers in the tables to a defined amount of decimal numbers (i.e. 2-3.). Especially in Table 3 this would be very helpful.

AR: Corrected as suggested

- You mention Figure 2 but further explanation, what 2A and 2B represents, is missing. Overall, I prefer bar-charts over pie-charts... this could also display the standard deviation. I leave it up to you, if you would like to change the style of presentation, but I would suggest it...

AR: Pie chart is replaced with bar plot.

-Why was smoking status included in the univariate analysis? Does it affect the activity of the arm in any way? I mean, we are not talking about wound healing problems but functionality, therefore i dont really understand the reason.

AR: This was actually an error which we apologize. Smoking was not included in the model

- I do not really understand the Tables well enough. I understand that you did a univariate analysis on the association of the activity of the injured arm with different PROMs and two different cutoff definitions. But I cant really wrap my head around the (un)adjusted beta which that displays the change in DASH when the activity level is increased by 100 minutes. So each patient has a certain amount of time in different activity levels and then check if the DASH is better if more time is spent in higher activity? And what specifically is the adjusted beta for activity? I think this requires a little more explanation for the reader to understand properly.

AR: We have now thoroughly revised the Table legends.

- I think, another limitation might be that the patients might be especially careful with the injured arm if a sensor is attached to it which will later be analyzed by a doctor. I can imagine that they are especially careful, so the doctor does not think, they are doing "too much"...

AR: We thank you for the comment. This is a probable source of bias, similar to Hawthorne effect. We have added many changes to the discussion and decided to leave this undiscussed.

- Generally, one of the main problems is that thresholds are not validated for the upper extremity. But I like your approach with taking both/all of the known one and comparing them.

AR: This is indeed a problem, which is, of course, a problem very hard to mitigate.

- I would appreciate 1-2 sentences discussing the objective vs. subjective outcomes/outcome measures for the patient. I personally think that it is important for the patient to be happy and have a good feeling about the overall outcome because he/she has to live with it. Objective outcome parameter are also necessary but should they overrule the subjective feeling of the patient?

AR: This is a very important aspect. We added a sentence to the fourth discussion paragraph. “While objective measures are essential for standardised assessment, treatment decisions must ultimately reflect patient-perceived recovery and quality of life, as these determine the lived impact of the outcome.”

Thank you very much for your work.

________________________________________

6. PL

Attachment Submitted filename: Responses_R1_AL.docx

# Editor report: Decision Letter 1

**DOI:** 10.1371/journal.pone.0352276.r003

**Authors:** Hans-Peter Simmen

8 Jun 2026

Association between patient-reported outcomes and accelerometer-based activity in

elderly patients 12 months after proximal humerus fracture. Results from two

randomized controlled trials

PONE-D-26-00155R1

Dear Dr. Reito,

After revision your paper is much more informative. Congratulations ! We’re pleased to inform you that your manuscript has been judged scientifically suitable for publication and will be formally accepted for publication once it meets all outstanding technical requirements.

Within one week, you’ll receive an e-mail detailing the required amendments. When these have been addressed, you’ll receive a formal acceptance letter and your manuscript will be scheduled for publication.

An invoice will be generated when your article is formally accepted. Please note, if your institution has a publishing partnership with PLOS and your article meets the relevant criteria, all or part of your publication costs will be covered. Please make sure your user information is up-to-date by logging into Editorial Manager at Editorial Manager® and clicking the ‘Update My Information' link at the top of the page. For questions related to billing, please contact billing support.

If your institution or institutions have a press office, please notify them about your upcoming paper to help maximize its impact. If they’ll be preparing press materials, please inform our press team as soon as possible -- no later than 48 hours after receiving the formal acceptance. Your manuscript will remain under strict press embargo until 2 pm Eastern Time on the date of publication. For more information, please contact onepress@plos.org.

Kind regards,

Hans-Peter Simmen, M.D., Professor of Surgery

Academic Editor

PLOS One

Additional Editor Comments (optional):

Reviewers' comments:

# Editor report: Acceptance letter

**DOI:** 10.1371/journal.pone.0352276.r004

**Authors:** Hans-Peter Simmen

PONE-D-26-00155R1

PLOS One

Dear Dr. Reito,

I'm pleased to inform you that your manuscript has been deemed suitable for publication in PLOS One. Congratulations! Your manuscript is now being handed over to our production team.

At this stage, our production department will prepare your paper for publication. This includes ensuring the following:

* All references, tables, and figures are properly cited

* All relevant supporting information is included in the manuscript submission,

* There are no issues that prevent the paper from being properly typeset

You will receive further instructions from the production team, including instructions on how to review your proof when it is ready. Please keep in mind that we are working through a large volume of accepted articles, so please give us a few days to review your paper and let you know the next and final steps.

Lastly, if your institution or institutions have a press office, please let them know about your upcoming paper now to help maximize its impact. If they'll be preparing press materials, please inform our press team within the next 48 hours. Your manuscript will remain under strict press embargo until 2 pm Eastern Time on the date of publication. For more information, please contact onepress@plos.org.

You will receive an invoice from PLOS for your publication fee after your manuscript has reached the completed accept phase. If you receive an email requesting payment before acceptance or for any other service, this may be a phishing scheme. Learn how to identify phishing emails and protect your accounts at https://explore.plos.org/phishing.

If we can help with anything else, please email us at customercare@plos.org.

Thank you for submitting your work to PLOS One and supporting open access.

Kind regards,

PLOS ONE Editorial Office Staff

on behalf of

Dr. Hans-Peter Simmen

Academic Editor

PLOS One
